# Noise-Robust Pulse Wave Estimation from Near-Infrared Face Video Images Using the Wiener Estimation Method †

**DOI:** 10.3390/jimaging9100202

**Published:** 2023-09-28

**Authors:** Yuta Hino, Koichi Ashida, Keiko Ogawa-Ochiai, Norimichi Tsumura

**Affiliations:** 1 Graduate School of Science and Engineering, Chiba University, Chiba 263-8522, Japantsumura@faculty.chiba-u.jp (N.T.); 2 Kampo Clinical Center, Hiroshima University Hospital, Hiroshima 734-8511, Japan; ikkandoo@gmail.com

**Keywords:** near-infrared, Wiener estimation method, pulse wave estimation

## Abstract

In this paper, we propose a noise-robust pulse wave estimation method from near-infrared face video images. Pulse wave estimation in a near-infrared environment is expected to be applied to non-contact monitoring in dark areas. The conventional method cannot consider noise when performing estimation. As a result, the accuracy of pulse wave estimation in noisy environments is not very high. This may adversely affect the accuracy of heart rate data and other data obtained from pulse wave signals. Therefore, the objective of this study is to perform pulse wave estimation robust to noise. The Wiener estimation method, which is a simple linear computation that can consider noise, was used in this study. Experimental results showed that the combination of the proposed method and signal processing (detrending and bandpass filtering) increased the SNR (signal to noise ratio) by more than 2.5 dB compared to the conventional method and signal processing. The correlation coefficient between the pulse wave signal measured using a pulse wave meter and the estimated pulse wave signal was 0.30 larger on average for the proposed method. Furthermore, the AER (absolute error rate) between the heart rate measured with the pulse wave meter was 0.82% on average for the proposed method, which was lower than the value of the conventional method (12.53% on average). These results show that the proposed method is more robust to noise than the conventional method for pulse wave estimation.

## 1. Introduction

It is noted that a part of research was presented Color and Imaging Conference 2022 [1]. Recently, there has been growing demand for monitoring technology in dark areas. Examples of applications of monitoring technology in dark areas include vital signs monitoring during sleep [2]. One method to achieve this is to attach contact-type measurement devices. However, the prolonged use of contact-type devices may cause discomfort to users. In addition, some people have difficulty in attaching contact-type devices; for example, newborns and people with burns [3,4]. Because of these disadvantages of contact-type devices, non-contact measurement methods are being proposed.

One non-contact measurement technique is the estimation of a pulse wave from face video images. By capturing subtle changes in the light reflected from the skin with a camera, pulse waves can be estimated [5,6]. From the estimated pulse wave, it is possible to obtain biometric information such as heart rate. Kurita et al. proposed a method for pulse wave estimation by separating RGB video images into melanin, hemoglobin, and shade components for analysis [7]. However, RGB cameras have problems in capturing video images and pulse wave estimations in low-light conditions. Near-infrared cameras have the advantage of being able to capture video images in low-light conditions. Garbey et al. proposed a method to measure pulse waves using a single-band medium wavelength infrared camera [8]. Zeng et al. proposed a method to estimate the pulse rate using a single-band near-infrared camera [9]. However, a single-band camera has the problem that the pulse wave estimation is affected by illumination in an environment with illumination fluctuation. Mitsuhashi et al. proposed a pulse wave estimation method that can be used in dark areas by using two-band near-infrared video images [10]. This method used two-band near-infrared video images to separate hemoglobin and shade components. By separating the components, the effect of illumination was removed and the pulse wave signal was estimated. However, this method does not consider noise for pulse wave estimation; thus, there is the issue with acquiring a distorted waveform when estimating the pulse wave signal. A distorted waveform may adversely affect the accuracy of physiological information obtained from pulse wave signals. In addition, hemoglobin absorption is weak in the near-infrared environment and estimated pulse wave signals are highly affected by noise [11,12].

There are some methods for denoising during pulse wave estimation [13,14]. However, these methods are deep-learning-based methods which can be complex in processing and difficult to implement. In addition, there are only a few types of large-scale datasets that include human biological information.

In this paper, therefore, we propose a method for estimating pulse waves in dark areas while considering the effect of noise. Our proposed method is a modified version of Mitsuhashi et al.’s method [10]. The Wiener estimation method was used for pulse wave estimation to consider noise. This estimation method can consider noise added to the video images by a linear operation. Therefore, it does not require complex processing techniques such as deep learning. Noise in images can be caused by thermal noise, illumination fluctuation or body motion. In this paper, we focus only on noise caused by thermal noise.

## 2. Skin Model in the Near-Infrared Environment

Light is classified into three categories: ultraviolet light, visible light and infrared light. Infrared light has a long wavelength range and is classified into near-infrared rays, mid-infrared rays and far-infrared rays according to its wavelength range. In this study, pulse wave estimation was performed using near-infrared video images in the wavelength range of approximately 780 nm to 1000 nm.

**Figure 1:**
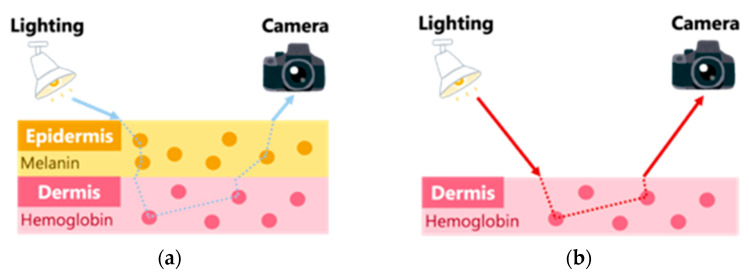
Skin model at different light wavelengths. (**a**) Visible light; (**b**) infrared light.

Human skin is a multilayered structure that can be divided into three main parts: the epidermis, dermis and subcutaneous tissue. The skin contains various pigments such as melanin, hemoglobin and bilirubin. Among these pigments, changes in melanin and hemoglobin have a significant effect on skin coloration. Melanin is found in the epidermis, whereas hemoglobin is found in the dermis, where capillaries are located. Kurita et al. [7] used a two-layer skin model composed of the epidermis and dermis. They simplified the skin model by assuming that the epidermis is a layer containing only melanin and the dermis is a layer containing only hemoglobin. This allows us to assume that the layer containing only melanin and the layer containing only hemoglobin are spatially independent. Therefore, under visible light illumination, human skin can be treated as a two-layer skin model consisting of a melanin layer and a hemoglobin layer, as shown in Figure 1a. In Kurita et al. [7], melanin and hemoglobin components were estimated using independent component analysis after removing shading components from the observed signal in the density space. In order to separate the melanin, hemoglobin and shade components in a visible light environment, a three-band image such as RGB image is required.

Kurita et al. [7] used an RGB camera; images obtained from an RGB camera contain only information obtained from visible light. On the other hand, near-infrared light has a longer wavelength than visible light. Therefore, it has a deeper penetration depth into the inside of a living body and is considered to be able to measure blood vessels deep inside the body, called microvessels, which exist even deeper than capillaries [15,16,17]. Therefore, it can be assumed that when light in the near-infrared environment enters the skin, reflection occurs only in the dermis, as shown in Figure 1b [9]. In this environment, no diffuse reflection occurs in the dermis. In order to separate the hemoglobin and shade components in the near-infrared environment, a two-band image is required. Therefore, the influence of the melanin component in the epidermis can be ignored in the near-infrared environment, whereas the melanin component is important in the visible light environment.

## 3. Hemoglobin and Shade Component Separation

### 3.1. Conventional Method

**Figure 2:**
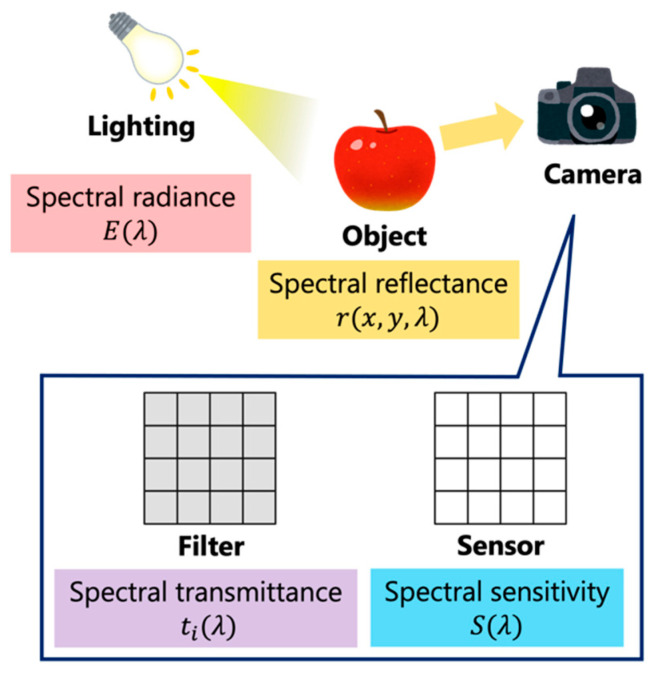
Process flow of Equation (1).

The pixel value $v_{i}$ when captured by the camera is expressed by the following equation:

(1) $$v_{i}=\int t_{i}\left(\lambda \right)E\left(\lambda \right)S\left(\lambda \right)r\left(x,y,\lambda \right)d\lambda ,\;i=1,\ldots ,m,$$

where $t_{i}(\lambda )$ is the spectral transmittance of the *i*th filter, E(λ) is the spectral radiance of the illumination, S(λ) is the spectral sensitivity of the sensor and r(x,y,λ) is the spectral reflectance of the object at coordinates (x,y). Equation (1) can be represented graphically as in Figure 2.

Expressing Equation (1) in matrix form, it can be expressed as follows:

(2) v=Fr,

where v is a vector of pixel values, F is a matrix summarizing spectral transmittance, spectral radiance and spectral sensitivity and r is a vector of object reflectance. This equation is also valid when replacing v with a vector of the negative logarithm of the pixel values and r with a vector of hemoglobin and shade components [9]. In the following sections, we will use the above replacement.

Equation (2) is the equation for the case where no noise is added. However, noise is added to the image during actual image capture due to thermal noise and other causes. Therefore, when noise is added, Equation (2) can be expressed as follows:

(3) v=Fr+n,

where n denotes noise.

When estimating r from v, the following equation is used for estimation:

(4) $$r=F^{-1}v.$$

It is noted that this estimation made by using simple inverse matrix (Equation (4)) is used as the conventional method in this paper. However, Equation (4) does not consider the noise added in Equation (3). Therefore, the estimated pigment components may differ significantly from the original ones.

### 3.2. Proposed Method

To estimate r from v after considering noise, the following matrix G can be used:

(5) $$\tilde{r}=Gv,$$

where $\tilde{r}$ is the pigment component estimated using the Wiener estimation method. It is desirable to minimize the error between the correct value r and the estimated value $\tilde{r}$. For this purpose, the mean squared error between r and $\tilde{r}$ is first calculated. The mean square error can be expressed as follows:

(6) $$MSE=\langle {\left(r-\tilde{r}\right)}^{T}(r-\tilde{r})\rangle ,$$

where ⟨⟩ denotes the ensemble mean for the pigment component vector. The estimation matrix that minimizes the mean squared error shown in Equation (6) is then expressed in Equation (7).

(7) $$G=R_{rv}R_{vv}^{-1},$$

where $R_{rv}$ denotes the cross-correlation matrix of r and v, and $R_{vv}$ denotes the autocorrelation matrix of v.

(8) $$R_{rv}=\langle rv^{T}\rangle ,$$

(9) $$R_{vv}=\langle vv^{T}\rangle ,$$

The autocorrelation matrix of r is expressed as in Equation (10).

(10) $$R_{rr}=\langle rr^{T}\rangle ,$$

Using this, Equation (7) can also be expressed as in Equation (11).

(11) $$G=R_{rr}F^{T}{\left(FR_{rr}F^{T}\right)}^{-1}.$$

In this case, we assume that noise is added to the images, as shown in Equation (3). As a result, the estimation matrix is given by following equation:

(12) $$G=R_{rr}F^{T}{\left(FR_{rr}F^{T}+R_{nn}\right)}^{-1},$$

where $R_{nn}$ denotes the autocorrelation matrix of the noise.

(13) $$R_{nn}=\langle nn^{T}\rangle ,$$

As described above, the Wiener estimation method gives the estimation matrix that minimizes the mean-square error between the correct and estimated values using a simple linear operation when the signal and noise statics are known [18].

## 4. Experimental Setup and Methods

### 4.1. Experimental Setup

**Figure 3:**
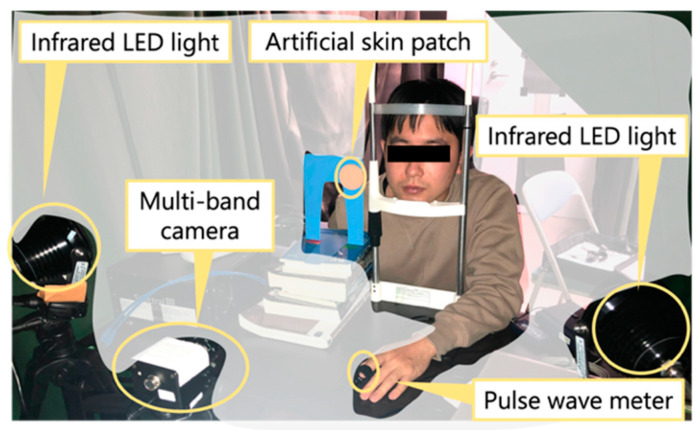
Experimental setup for capturing face video images.

We performed the experiments on a lab test bench setup. The experimental setup is shown in Figure 3. Three male subjects in their 20 s participated in this experiment. In a darkroom, the subject was illuminated with infrared LED lights (SA6-IR, EnergyPower, Hong Kong, China). The wavelength of this light is 850 nm. Face video images were captured for 30 s using a multi-band near-infrared camera (Spectral Devices, MSC2-BIO-1-A) at 66.5 frames per second. Imaging was performed once for each subject. Each video image was saved as a still image, frame by frame to avoid the effects of compression. A total of 1995 frames of images were obtained for each subject. This camera had four wavelength bands centered at 735 nm, 800 nm, 865 nm and 930 nm. The resolution of each image was 512 × 512 pixels. The artificial skin patch was captured at the same time. The artificial skin patch was used to obtain the autocorrelation matrix $R_{nn}$ of the noise added to the captured video images. Simultaneously with the imaging, a pulse wave signal was measured by attaching a photoelectric pulse wave meter (Procomp Infiniti, Thought Technology, Montreal, Canada) to the tip of the index finger of the subject’s left hand. The subjects were instructed to place their chin on a chin rest, which minimized the subject’s head motion as much as possible. In addition, the subjects were instructed to move their head and finger as little as possible during imaging.

### 4.2. Calculation Autocorrelation Matrixes

As described in Section 3, the noise autocorrelation matrix $R_{nn}$ is required when using the Wiener estimation method. To obtain the noise autocorrelation matrix, it is necessary to obtain the noise added to the captured video images. In this study, the noise added to the video images was obtained by simultaneously capturing an artificial skin patch with the subject’s face. Ideally, the pixel values of artificial skin patch do not change because the surface condition of the artificial skin patch does not change over time. However, the pixel values of the artificial skin patch changed due to the influence of noise. This change in pixel value was used to determine the magnitude of the noise.

**Figure 4:**
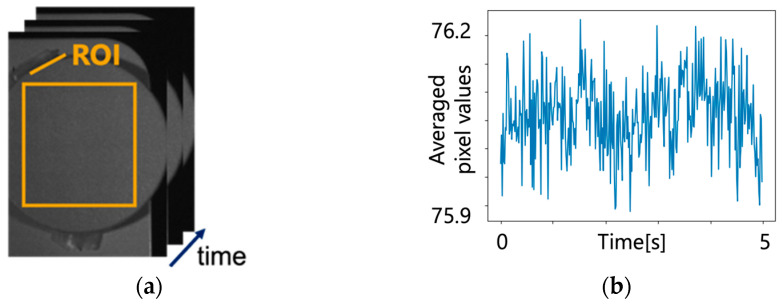
Near-infrared video images of artificial skin patch. (**a**) Video images of artificial skin patch; (**b**) averaged pixel values of (**a**).

The ROI (region of interest) was set for the image of the artificial skin patch, as shown in Figure 4, and the temporal variation of the averaged pixel value was calculated within the ROI. The standard deviation was also calculated on the temporal variation of the averaged pixel value. The above process was performed on the two bands used for imaging and the larger standard deviation was used in subsequent procedures. Then, Gaussian noise was generated by setting the mean value to 0 and the standard deviation to the obtained value. Finally, the autocorrelation matrix of the noise $R_{nn}$ was obtained using this generated noise.

The autocorrelation matrix of the skin pigment $R_{rr}$ is also required when using the Wiener estimation method. To calculate $R_{rr}$, a value of r is required. In this study, the value of r obtained using the conventional method (Equation (4)) was used. The pigment component r consists of a hemoglobin component and a shade component, and both the hemoglobin and shade component were calculated as the average of the values obtained from each of the three subjects. When calculating $R_{rr}$, the hemoglobin component values varied within the range of values obtained from three subjects, while the shade component values were fixed. This reflects the fact that the hemoglobin component fluctuated with time because of the blood, while the shade component did not. With the above settings of r, $R_{rr}$ was calculated.

### 4.3. Acquisition of the Original Pulse Wave Signal and Signal Processing

**Figure 5:**
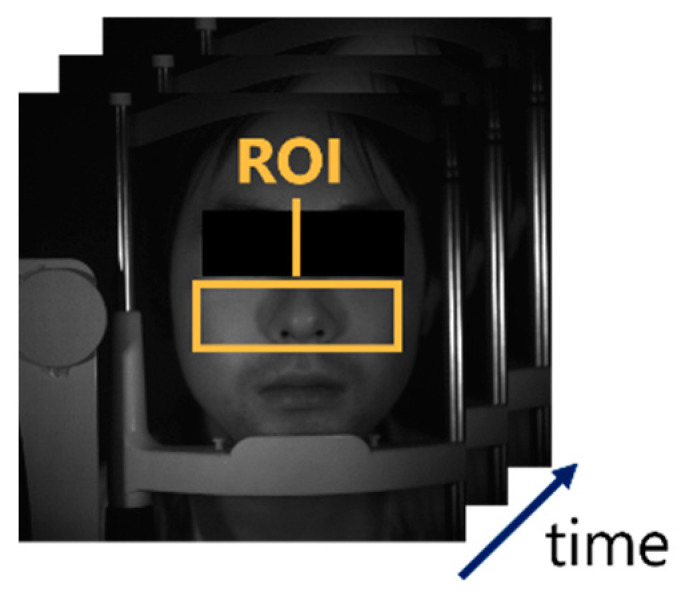
Near-infrared face video images and ROI setting.

The temporal variation in the averaged pixel values in the ROI was analyzed by selecting two bands from the multi-band near-infrared face video images taken under the imaging environment described in Section 4.1 and setting the ROI as shown in Figure 5. The nose and cheeks were selected as the ROI [19]. In a vertical direction, the ROI was set in the range shown in Figure 5 to avoid the eye blink and lip motion. The ROI size was set as large as possible within the above range because a larger ROI size reduces noise [20]. In the current study, two-band images were used: one with a central wavelength of 800 nm and the other with a central wavelength of 930 nm. These two wavelengths were selected based on a previous study [10]. By using the temporal variation in the acquired average pixel values in Equations (4) and (5), respectively, the estimation results of the original pulse wave signal by the conventional and proposed methods could be obtained.

Detrending [21] was performed on the original pulse wave signal. A bandpass filter was then applied. The frequency range transmitted by the bandpass filter was set to [0.75, 4.0] Hz [22,23]. The upper peak points were detected by finding the local maximum values for each waveform in the bandpass-filtered pulse wave signal. The peak points are used to estimate the heart rate. The heart rate can be calculated by using the interval between adjacent peak points, called the *RR interval.* The heart rate can be calculated by dividing 60 by $\bar{RR_{interval}}$ (the average of *RR interval* in the signal), as shown in Equation (14).

(14) $$HR=\frac{60}{\bar{RR_{interval}}}$$

### 4.4. Evaluation Metrics

In this paper, the correlation coefficient, SNR (signal to noise ratio) and AER (absolute error rate) were used as evaluation metrics for pulse wave estimation. To calculate *SNR*, the pulse wave signals were Fourier-transformed to obtain the “*Signal*” component (0.5–15 Hz) and the “*Noise*” component (frequencies after 15 Hz) [24]. The “*Signal*” and “*Noise*” components were used to calculate the *SNR* from Equation (15).

(15) $$SNR=20\log_{10}\left(\frac{Signal}{Noise}\right)\;\left[dB\right].$$

The *AER* between the estimated heart rate and the heart rate obtained using a pulse wave meter was determined [10]. *AER* is expressed by the following equation:

(16) $$AER=\frac{\left|HR_{GT}-HR_{EV}\right|}{HR_{GT}}\times 100.$$

where $HR_{GT}$ is the ground truth of the heart rate obtained using a pulse meter and $HR_{EV}$ is the estimated heart rate obtained from the pulse wave estimated by the conventional or proposed method.

## 5. Results

### 5.1. Original Pulse Wave Signals

**Figure 6:**
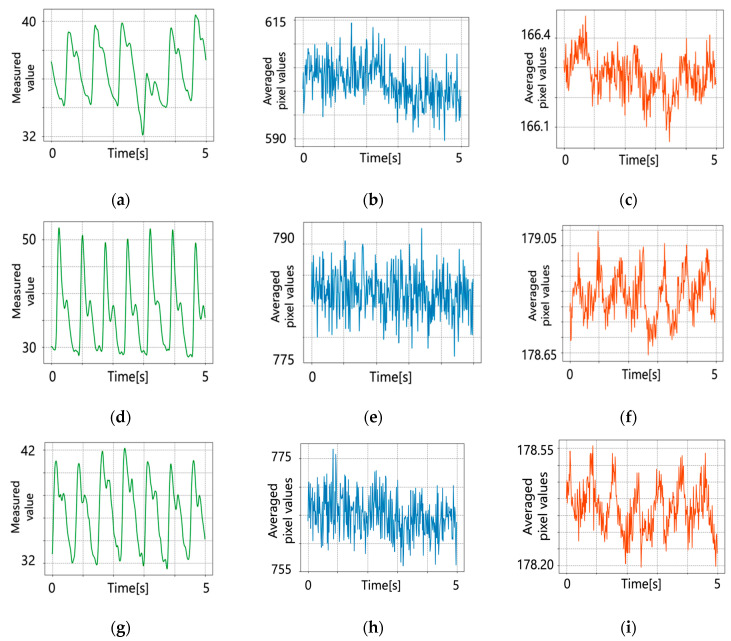
Original pulse wave signals. (**a**) Pulse wave meter (subject 1); (**b**) conventional method (subject 1); (**c**) proposed method (subject 1); (**d**) pulse wave meter (subject 2); (**e**) conventional method (subject 2); (**f**) proposed method (subject 2); (**g**) pulse wave meter (subject 3); (**h**) conventional method (subject 3); and (**i**) proposed method (subject 3).

Figure 6 shows the original pulse wave signals obtained by applying the conventional and proposed methods to two-band near-infrared images and pulse wave signals obtained using a pulse wave meter. The correlation coefficient was calculated between the estimated pulse wave signals and the pulse wave signal obtained using a pulse wave meter. SNR was calculated from the pulse wave signals. Based on the results of the correlation coefficient and SNR (Table 1), the proposed method provides a stronger correlation than the conventional method. The results on SNR show that the proposed method can estimate pulse wave signals robustly regarding noise.

### 5.2. After Signal Processing

**Figure 7:**
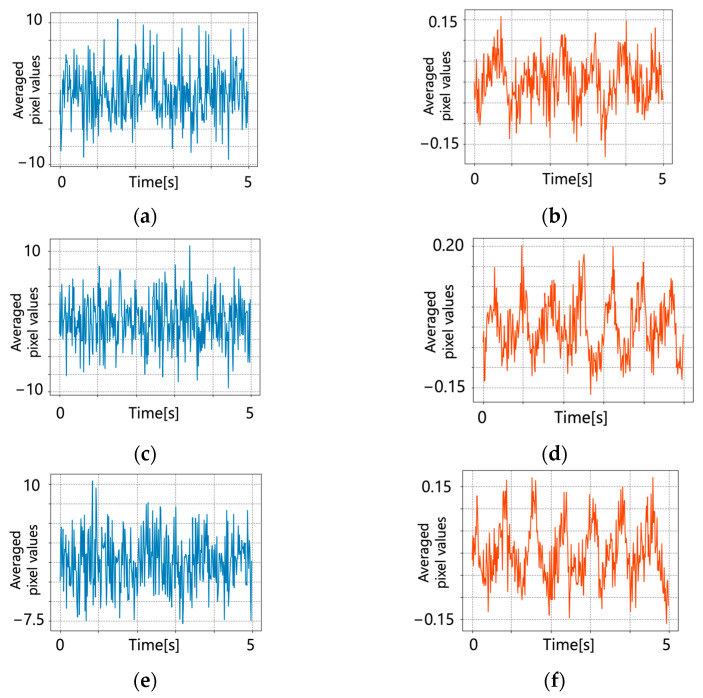
Pulse wave signals after detrending. (**a**) Conventional method (subject 1); (**b**) proposed method (subject 1); (**c**) conventional method (subject 2); (**d**) proposed method (subject 2); (**e**) conventional method (subject 3); (**f**) proposed method (subject 3).

**Figure 8:**
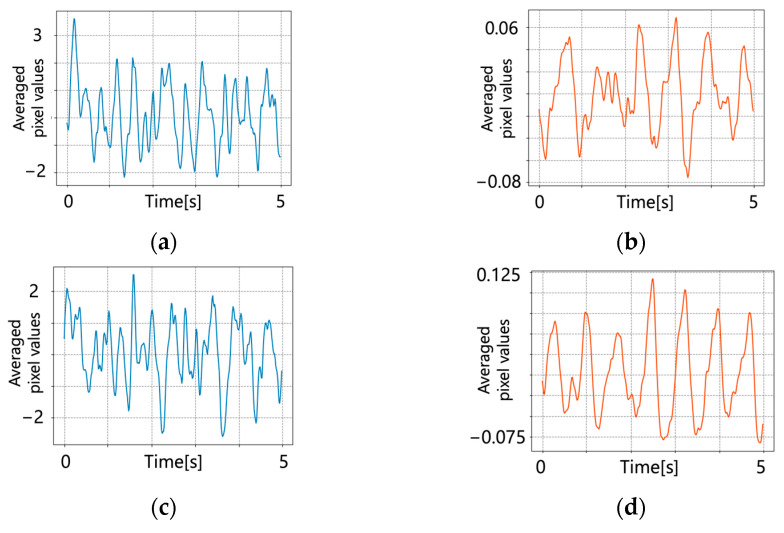

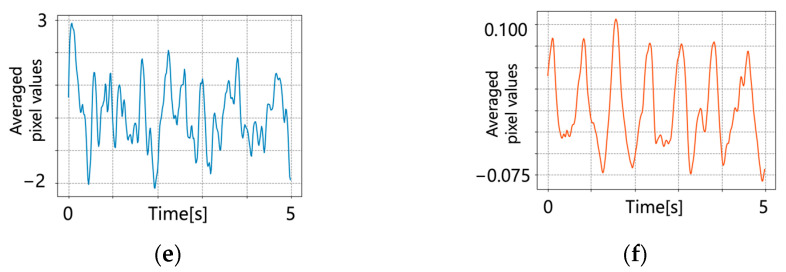
Pulse wave signals after bandpass filtering. (**a**) Conventional method (subject 1); (**b**) proposed method (subject 1); (**c**) conventional method (subject 2); (**d**) proposed method (subject 2); (**e**) conventional method (subject 3); (**f**) proposed method (subject 3).

Detrending was performed on the original pulse wave signal and the results are shown in Figure 7. A bandpass filter was applied. Figure 8 shows the results of applying the bandpass filter.

Correlation coefficients were calculated between each pulse wave signal and the pulse wave signal obtained using a pulse wave meter. SNR was calculated from each estimated pulse wave signal. Furthermore, the heart rate was estimated from each bandpass-filtered pulse wave signal, and AER was calculated between the estimated heart rate and the heart rate obtained from the pulse wave meter. The heart rate was estimated only from the bandpass-filtered pulse wave signals because the waveform after bandpass filtering had clearer peak points. Table 2 shows the results for the correlation coefficient, SNR and AER after each signal processing; the combined use of detrend and bandpass filtering makes the pulse wave signal robust to noise and improves the accuracy of heart rate estimation using the pulse wave signal. The pulse wave signal estimated using the proposed method showed a stronger correlation coefficient with the pulse wave signal obtained from a pulse wave meter. Furthermore, for the SNR and AER the results showed that the proposed method can achieve noise-robust pulse wave estimation and highly accurate heart rate estimation.

## 6. Discussion

The advantage of the proposed method is that it shows robustness to noise at the point of the original pulse wave signal, as shown in Figure 6. This makes it possible to estimate the pulse wave using the proposed method even when the pulse wave is buried in noise using only a bandpass filter, as shown in Figure 8a. The accuracy of heart rate estimation differed among subjects (Table 1 and Table 2). This may be due to slight differences in skin thickness [25] and the position of blood vessels among the subjects, which affects the accuracy of pulse wave estimation.

**Figure 9:**
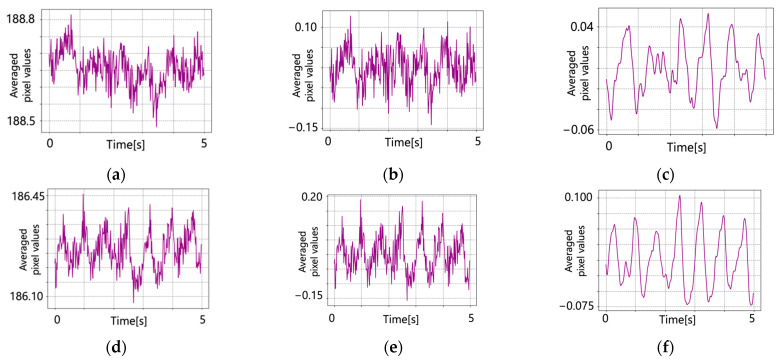

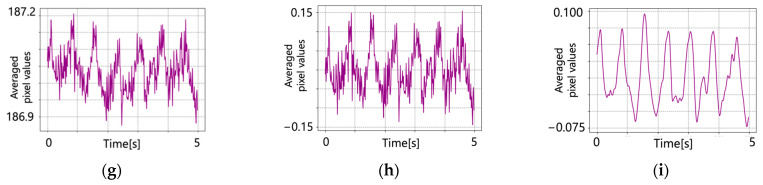
Pulse wave signals when changing the configuration of the pigment component values used for $R_{rr}$. (**a**) Original pulse wave signal (subject 1); (**b**) pulse wave signal after detrending (subject 1); (**c**) pulse wave signal after applying bandpass filtering (subject 1); (**d**) original pulse wave signal (subject 2); (**e**) pulse wave signal after detrending (subject 2); (**f**) pulse wave signal after applying bandpass filtering (subject 2); (**g**) original pulse wave signal (subject 3); (**h**) pulse wave signal after detrending (subject 3); and (**i**) pulse wave signal after applying bandpass filtering (subject 3).

In this paper, the sample size was small (three subjects). In order to provide sufficient discussion with a small number of data, we describe below a discussion of the relationship between the calculation method of the autocorrelation matrix *R_rr_* used in the Wiener estimation method and the results. As described in Section 4.2, when calculating the autocorrelation matrix $R_{rr}$ for the pigment components, the hemoglobin and shade component values were the average values for all subjects obtained using the conventional method (Equation (4)). Here, we show the results obtained when calculating $R_{rr}$ by utilizing the values of hemoglobin and shade components for each individual subject. Figure 9 shows the original pulse wave signal and the pulse wave signal after signal processing (detrend and bandpass filter) under the above condition settings. Table 3 shows the correlation coefficient with the pulse wave signal obtained from the pulse wave meter, the SNR of each estimated pulse wave signal and the AER between the estimated heart rate and the heart rate obtained from the pulse wave meter. Comparing Figure 9 with Figure 6, Figure 7 and Figure 8, although a change in the values of the vertical axis occurred in the original pulse wave signals, there was almost no effect on the approximate shape of the original pulse wave signals. Comparing Table 3 with Table 1 and Table 2, the SNR was lowest in the case of subject 1, regardless of the method used to set the value of $R_{rr}$. The correlation coefficient, SNR and AER did not change significantly. These results indicate that the method of setting the $R_{rr}$ value does not significantly influence the pulse wave estimation results. This may be due to the similarity between the values of hemoglobin and shade components obtained from each subject and the average of the component values for all subjects. However, since the subjects in this experiment had a narrow range of age, race, gender and sex, the method used to set the $R_{rr}$ value is not considered to have a significant effect on the results. Further experiments will be performed with subjects of various ages, races and genders to further examine the individual differences in the accuracy of pulse wave estimation and the effect of the $R_{rr}$ value setting method on accuracy in the future.

## 7. Conclusion and Future Works

In this study, we proposed a noise-robust pulse wave estimation method for near-infrared face video images using the Wiener estimation method. We compared the proposed method with a conventional method in a near infrared environment. For pulse wave estimation in the near-infrared environment, pulse waves can be obtained by separating hemoglobin and shade components in two-band face video images. While the conventional method uses the inverse matrix of the pigment components (hemoglobin and shade component) vectors, the Wiener estimation method approximates the inverse matrix used in the conventional method by using the autocorrelation matrix of the pigment components and the autocorrelation matrix of the noise added to video images. Therefore, the estimation matrix obtained by the Wiener estimation method can consider noise, whereas the conventional method does not have any information about noise in the matrix used for component separation. The Wiener estimation method is a linear operation and can consider noise without the need for complex processing methods such as deep learning.

The pulse wave signal was estimated using the proposed method and was compared with the one estimated using the conventional method. As evaluation metrics, we used the correlation coefficient between the pulse wave signal obtained from a pulse wave meter and the estimated pulse wave signal, the SNR of each estimated pulse wave signal and the AER between the estimated heart rate (only the estimated pulse wave signals that had been detrended and bandpass filtered) and the heart rate obtained from the pulse wave meter. In the comparison, experimental results showed that the combination of the proposed method and signal processing (detrending and bandpass filtering) increased the SNR by more than 2.5 dB compared to the conventional method and signal processing. The correlation coefficient between the pulse wave signal measured using the pulse wave meter and the estimated pulse wave signal was 0.48 on average for the proposed method and 0.18 on average for the conventional method, indicating a stronger correlation with the proposed method. Furthermore, the AER with the heart rate measured using the pulse wave meter averaged 0.82% for the proposed method and 12.53% for the conventional method, indicating that the pulse wave estimated using the proposed method can be used to estimate the heart rate with high accuracy.

Although the results in the near-infrared environment were better than those obtained with the conventional method, it is necessary to verify the use of this method in environments other than the near-infrared environment, such as in RGB photography. It is also necessary to verify the method when the noise is larger than the noise added in this experiment. In this paper, only the noise caused by thermal noise was considered. However, for practical use, it will be necessary to consider other types of noise in the future.

## Figures and Tables

**Table 1:** Comparison of conventional and proposed methods (original pulse wave signals).

Subjects	Methods	Correlation Coefficient	SNR [dB]
Subject 1	Conventional	−0.004	−8.1
	Proposed	−0.027	−4.8
Subject 2	Conventional	−0.020	−8.4
	Proposed	−0.078	−4.6
Subject 3	Conventional	−0.004	−8.2
	Proposed	0.059	−3.0

**Table 2:** Correlation coefficient, SNR and AER results after each signal processing.

Subjects	Methods	Signal Processing	Correlation Coefficient	SNR [dB]	AER [%]
Subject 1	Conventional	Detrend	0.027	−8.8	-
		Detrend and bandpass filter	0.109	−5.5	20.6
	Proposed	Detrend	0.342	0.3	-
		Detrend and bandpass filter	0.506	3.3	0.90
Subject 2	Conventional	Detrend	0.096	−8.8	-
		Detrend and bandpass filter	0.187	2.1	7.68
	Proposed	Detrend	0.353	−4.8	-
		Detrend and bandpass filter	0.411	5.6	0.06
Subject 3	Conventional	Detrend	0.090	−2.9	-
		Detrend and bandpass filter	0.246	2.1	9.31
	Proposed	Detrend	0.332	−5.1	-
		Detrend and bandpass filter	0.517	4.7	1.50

**Table 3:** Correlation coefficient, SNR and AER results after changing $R_{rr}$ configuration.

Subjects	Methods	Signal Processing	Correlation Coefficient	SNR [dB]	AER [%]
Subject 1	Proposed	Original pulse	−0.027	−4.8	-
		Detrend	0.342	0.3	-
		Detrend and bandpass filter	0.506	3.3	0.90
Subject 2	Proposed	Original pulse	−0.078	−4.6	-
		Detrend	0.353	−4.8	-
		Detrend and bandpass filter	0.411	5.6	0.06
Subject 3	Proposed	Original pulse	0.059	−3.0	-
		Detrend	0.332	−5.1	-
		Detrend and bandpass filter	0.517	4.7	1.50

## Data Availability

The data presented in this study are available on request from the corresponding author. These data are not publicly available due to ethical restrictions.
